# Enrichment of G‐to‐U Substitution in SARS‐CoV‐2 Functional Regions and Its Compensation via Concurrent Mutations

**DOI:** 10.1002/jmv.70353

**Published:** 2025-04-18

**Authors:** Xierzhatijiang Sulaiman, Yan Han, Sheng Liu, Kailing Li, Marissa Shang, Lei Yang, Kenneth White, Yong Zang, Jikui Shen, Jun Wan

**Affiliations:** ^1^ Department of Medical and Molecular Genetics Indiana University School of Medicine Indianapolis Indiana USA; ^2^ Department of Biostatistics & Health Data Science Indiana University School of Medicine Indianapolis Indiana USA; ^3^ Indiana University Simon Comprehensive Cancer Center Indiana University School of Medicine Indianapolis Indiana USA; ^4^ Department of BioHealth Informatics Indiana University School of Informatics and Computing at IUPUI Indianapolis Indiana USA; ^5^ Queen's University Kingston Ontario Canada; ^6^ Center for Computational Biology and Bioinformatics Indiana University School of Medicine Indianapolis Indiana USA; ^7^ Herman B Wells Center for Pediatric Research, Department of Pediatrics Indiana University School of Medicine Indianapolis Indiana USA; ^8^ The Wilmer Eye Institute Johns Hopkins University School of Medicine Baltimore Maryland USA

**Keywords:** C‐to‐U deamination, coronavirus, evolution, G‐to‐U substitution, genetics, mutation, virus classification

## Abstract

We surveyed single nucleotide variant (SNV) patterns from 5 903 647 complete SARS‐CoV‐2 genomes. Among 10 012 SNVs, APOBEC‐mediated C‐to‐U (C > U) deamination was the most prevalent, followed by G > U and other RNA editing‐related substitutions including (A > G, U > C, G > A). However, C > U mutations were less frequent in functional regions, for example, S protein, intrinsic disordered regions, and nonsynonymous mutations, where G > U were over‐represented. Notably, G‐loss substitutions rarely appeared together. Instead, G‐gain mutations tended to more frequently co‐occur with others, with a marked preference in the S protein, suggesting a compensatory mechanism for G loss in G > U mutations. The temporal patterns revealed C > U frequency declined until late 2021 then resurged in early 2022. Conversely, G > U steadily decreased, with a pronounced drop in January 2022, coinciding with reduced COVID‐19 severity. Vaccinated individuals exhibited a slightly but significantly higher C > U frequency and a notably lower G > U frequency compared to the unvaccinated group. Additionally, cancer patients had higher G > U frequency than general patients during the same period. Interestingly, none of the C > U SNVs were uniquely identified in 2724 environmental samples. These findings suggest novel functional roles of G > U in COVID‐19 symptoms, potentially linked to oxidative stress and reactive oxygen species, while C > U remains the dominant substitution, likely driven by host immune‐mediated RNA editing.

## 1 Introduction

Understanding the mutational bias and their driving forces in SARS‐CoV‐2 is crucial for the prediction for the trajectory of virus evolution [1, 2, 3] and vaccine designs. Thus, measuring or surveying the overall mutation trend enables the monitoring of viral dynamics. One approach to tracking these trends is to analyze the distribution of mutations based on the 12 common substitution types, for example, C > A/G/U representing C‐to‐A/C‐to‐G/C‐to‐U, G > A/C/U, and so on. Several studies have documented a strong bias toward C > U transitions in the SARS‐CoV‐2 genome [4, 5, 6, 7], primarily attributed to APOBEC (the apolipoprotein‐B mRNA editing enzyme, catalytic polypeptide‐like protein) mediated C > U RNA deamination by the host participates different viral mutation profiles [7, 8, 9, 10]. Mutations in prokaryote and eukaryote typically favor transitions over transversions, with C > U deamination likely being a contributing factor [7, 8]. APOBEC enzymes, a family of cytidine deaminases involved in RNA editing, are known to influence viral mutation profiles, including those of SARS‐CoV‐2 [11, 12, 13]. This family has seven members, three APOBEC1, APOBEC3A, and APOBEC3G, deaminate certain cellular single‐stranded RNA (ssRNA) targets to cause C‐to‐U editing [11, 14]. These enzymes catalyze the conversion of cytosine to uracil (C > U) in single‐stranded RNA, a process commonly observed in viral genomes. In both prokaryotes and eukaryotes, transition mutations (purine‐to‐purine or pyrimidine‐to‐pyrimidine changes) are generally favored over transversions, with APOBEC‐driven C > U deamination contributing significantly to this trend. To this end, the mutated human adenosine deaminase acting on RNA (ADAR)2 deaminase domain is forced to inactive RNA‐targeting clustered regularly interspaced short palindromic repeats (CRISPR)‐Cas13b (dCas13b). C > U deamination is also involved in many pathophysiological procedures, including the development of cancer, viral evolution and mutation [15, 16, 17, 18, 19]. This RNA editing is originally a partial mechanism to generate protein diversity and transcript repair, possibly also helps protect from viral infections by restricting reverse transcription and replication [14, 20, 21, 22]. However, in the case of SARS‐CoV‐2, the C > U deamination seems to be acting as driving force of its evolution through making up the most dominant substitution type in the SNV pool [8, 9, 10, 23]. Also, divergence sites RAT23G and SARS‐CoV‐2 are predominantly C > U mutations [24]. These findings implicate C > U mutation mediated by RNA editing could benefit SARS‐CoV‐2 adaptation functionally. For examples, experimental research has showed that wild‐type APOBEC3 greatly promotes viral replication/propagation, suggesting that SARS‐CoV‐2 utilizes the APOBEC‐mediated mutations for evolutionary fitness [25]. However, due to limited data resources used in previous reports, details about the distribution of C > U deamination is unclear and may not reflect the true viral evolutions. Despite APOBEC's recognized role in RNA editing, its impact on SARS‐CoV‐2 evolution remains underexplored. To determine the directionality of selection for APOBEC‐mediated C > U mutations in SARS‐CoV‐2 evolution, especially whether C > U mutation has adaptive advantages for SARS‐CoV‐2, we used large scale of SARS‐CoV‐2 genome data and conducted an in‐depth survey of the distribution of found SARS‐CoV‐2 SNVs in different settings with genomic locations to investigate their preference of substitution type and functional implications. For example, SNVs in S protein could alter the ACE2‐binding affinity, and reduce the neutralizing activity of antibodies, and also make up the most variant defining mutations [26]. Intrinsically disordered regions (IDRs) make up 28% of S1 subunit of spike protein, harboring major variant defining mutations, and are enriched with antigenic sites [27, 28]. Substitution types of concurrent mutations, where two mutations occur simultaneously, were worth investigating as well [29]. Additionally, we examined the temporal trend of substitutions in SNV pools across different time points and major variants. It's reported that increase of C > U mutations correlate with enhanced inflammatory response [30]. Therefore, we also analyzed how different host immune environment influence substitution patterns by comparing SNVs from human hosts with known vaccination status, and those collected from environment.

## 2 Methods

### 2.1 Data Source

We collected SNV data from 5 903 647 high coverage complete genomic sequences from the GISAID database [31, 32] as of February 29, 2024. A total of 79 343 SNVs covering nearly all genomic positions (29 700 out of 29 993 nts) were identified through sequence alignments with the reference genome, Wuhan‐Hu‐1 (NCBI: NC_045512.2). Wuhan‐Hu‐1 represents the full‐length genome sequence of the SARS‐CoV‐2 detected in China in December 2019 [33] and has been widely used as standard reference. Among them, 10 012 SNVs were identified at 8920 base sites with a frequency exceeding 0.01% of all samples and were selected for further analysis. SNVs were also detected from 2724 samples collected from the environmental specimens (nonbiological) from the GISAID database. IDR information was collected from DisProt database [34].

### 2.2 Data Analysis

We set up a cutoff of 0.01% overall frequency for all SNVs. The remaining 10 012 SNVs were divided into 12 substitution types, A > C/G/U, C > A/G/U, G > A/C/U, U > A/C/G. For comparison of these 12 substitution types across the whole SARS‐CoV‐2 genome, we first calculated their frequency distribution in the way of

(1) $$F_{i>j}=\frac{M_{i>j}}{M_{\mathrm{total}}},$$

where *i* and *j* represent the nucleotide bases A, C, G, and U. *M*_*i>j*_ is the number of base change types, *i* > *j*, for all samples in the group of study, and *M*_total_ refers to the total number of detected substitutions. Considering the varying proportions of A, C, G, and U in the viral genome, we defined a mutation ratio to compare observed mutation frequency, *M*_*i>j*_ */N*_*i*_, to a theoretical average, *M*_total_ */N*_total_ such as

(2) $$\mathrm{Mutation}\;\mathrm{ratio}=F_{i>j}/\frac{N_{i}}{N_{\mathrm{total}}},$$

where *N*_*i*_ denotes the number of nucleotides with the reference base *i*, whereas *N*_total_ is the total number of nucleotides in the genome. The mutation ratio greater than 1/3 for a specific substitution type suggests its preference in the genome, given that a nucleotide can potentially mutate into any of the other three nucleotide types.

To further estimate mutation preferences within a specific group of interest, such as genomic regions, non‐synonymous mutations, or time periods, we calculated the fold enrichment (FE) score in the way of

(3) $$\mathrm{FE}(i>j)=\frac{m_{i>j}}{m_{\mathrm{total}}}/F_{i>j},$$

where *m*_*i*__*>*__*j*_ and *m*_total_ represent the total number of *i* > *j* mutation and nucleotides in selected group, respectively, while *F*_*i*>*j*_ is *i* > *j* mutation frequency in the background for comparison. Specifically, we compared substitution frequency for individual genes or time points to that observed across the whole genome. Fisher's exact test was applied to calculate *p* value and implemented using the command fisher. test() in R. Using G > U mutations in the S protein as an example, two columns in 2 × 2 contingency table include categories of mutations: G > U mutations (category 1) and all other mutations (category 2), while two rows correspond to occurrences of these mutations in the S protein (group 1) and across other proteins (group 2), respectively. For IDR and non‐synonymous mutations, we compared mutation frequencies within IDR or non‐synonymous mutations to those in corresponding genes.

We also investigated the patterns for concurrent mutations [29]. The concurrent ratio of two SNV sites, A and B, is defined as

(4) $$R\;\left(A,B\right)=\frac{|A\cap B|}{\min\;(|A|,|B|)},$$

where |A ∩ B| is the number of samples presenting SNVs at both sites, A and B, whereas min(|A|, |B|) represents the minimum number of samples bearing mutations at either A or B or both.

To assess whether nucleotide substitution pairs tend to occur simultaneously within identified concurrent pairs, we calculated the FE by comparing their observed frequencies to the expected frequencies

(5) $$\mathrm{FE}\left(S_{i>j},S_{k>m}\right)=\frac{O(S_{i>j},S_{k>m})}{\mathrm{En}(S_{i>j},S_{k>m})}/\frac{P_{0}}{N_{0}\times (N_{0}-1)/2},$$

where $S_{i>j}\;\mathrm{or}\;S_{k>m}$ represents a specific nucleotide substitution, *i* > *j* or *k* > m, $O(S_{i>j},S_{k>m})$ denotes the observed number of concurrent pairs of $S_{i>j}\;\mathrm{and}\;S_{k>m}$, *P*_*0*_ is the total number of concurrent pairs identified, *N*_0_ refers to the number of SNVs within *P*_0_ pairs, and $\mathrm{En}(S_{i>j},S_{k>m})$ is the expected numbers of substitutions pairs $S_{i>j}\;\mathrm{and}\;S_{k>m}$, calculated by

(6) $$\mathrm{En}(S_{i>j},S_{k>m})=\left\{\begin{array}{l} n(S_{i>j})\times (n(S_{k>m})-1)/2,i=k\;\mathrm{and}\;j=m\\ n(S_{i>j})\times n(S_{k>m}),\mathrm{otherwise}, \end{array}\right.$$

where $n(S_{i>j})$ and $n(S_{k>m})$ are the respective counts of substitutions appearing in concurrent mutation pairs, $S_{i>j}\;\mathrm{and}\;S_{k>m}$. Fisher's exact test was taken to determine whether the observed frequency of a concurrent mutation pair significantly deviates from what would be expected by chance.

## 3 Results

### 3.1 C > U and G > U Were Predominant Substitutions

**Figure 1:**
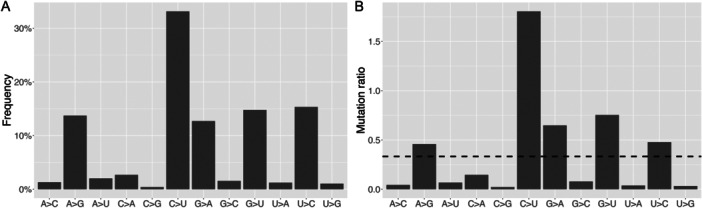
Statistics of 12 substitution types among all SNVs across the whole genome. (A) Distribution of frequencies. (B) Mutation ratio normalized by reference nucleotide counts across the genome. The line represents the expected ratio.

Among 10,012 SNVs identified in at least 0.01% of patient samples, C > U was the most prevalent substitution type (33.2%), followed by U > C (15.3%), G > U (14.8%), A > G (13.7%), and G > A (12.7%) (Figure 1A). To account for the varying proportions of A, C, G, and U within the genome, we further normalized mutation frequencies based on the ratios of the reference nucleotides across the genome (Equation 1). A similar pattern was observed (Figure 1B), where the mutation ratios of C > U, G > U, G > A, U > C, and A > G, all exceeding the expected baseline of 1/3, based on the theoretical assumption that each reference nucleotide can mutate into any one of the other three nucleotides.

### 3.2 G > U Was Enriched in the S Protein, IDRs, and Non‐Synonymous Mutations

**Figure 2:**
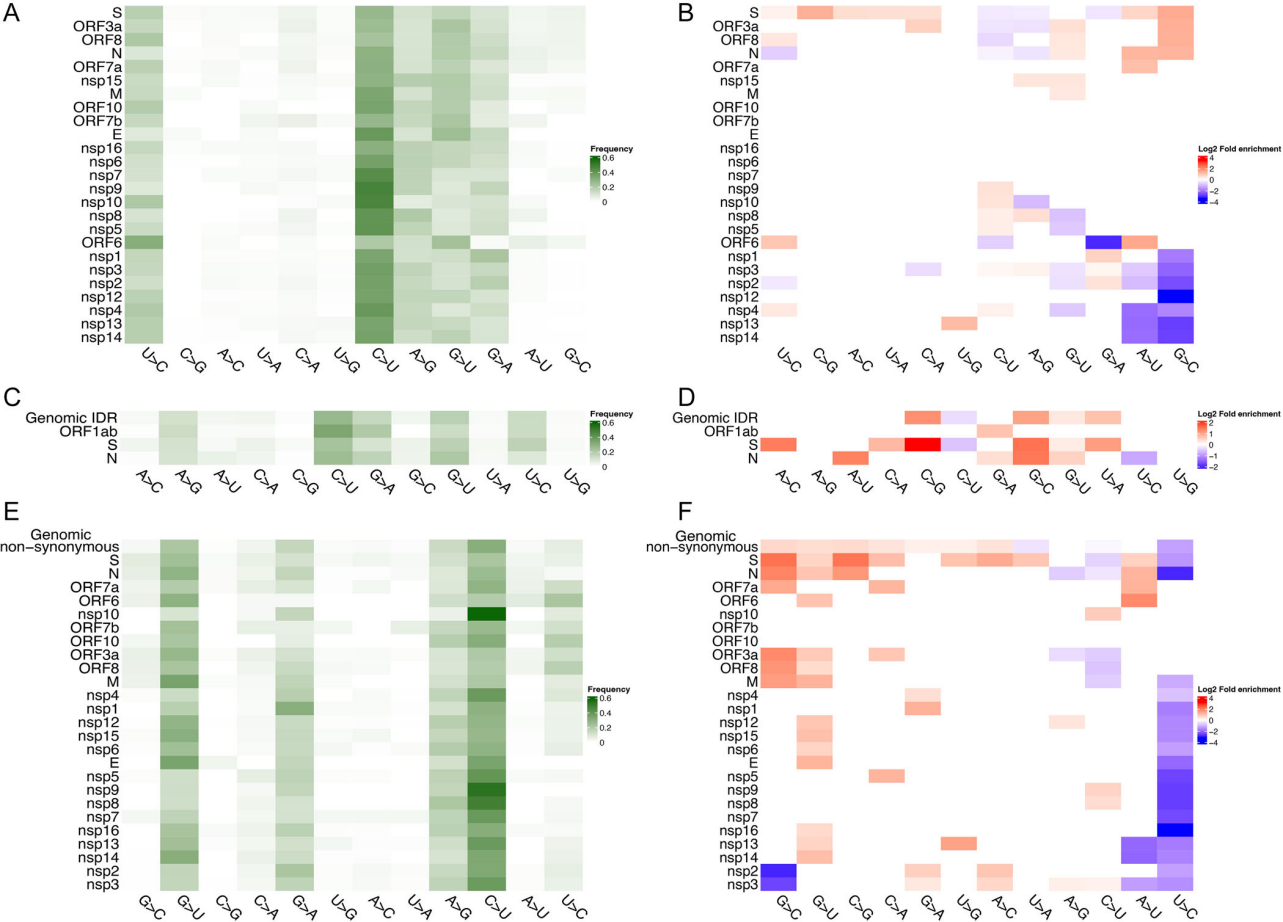
Nucleotide substitutions within individual structural areas of viral genome and IDRs or focused solely on nonsynonymous mutations. (A) Mutation frequencies in genes including open reading frame 1a and 1ab (ORF1a/ORF1ab) encoding nsp1 to nsp16, where darker color indicates higher frequency. (B) FE based on comparison to genome‐wide frequencies, where the FE values are log_2_ transformed, and red represents a frequency higher than the genome level while blue indicates a lower frequency. FE values with *p* > 0.05 were marked as 1. (C, D) Frequencies and FE in IDRs. (E, F) Frequencies and FE of non‐synonymous mutations in each gene.

Given the critical role of the S protein in determining the pathogenicity and immunogenicity of SARS‐CoV‐2, as well as its significant influence on the evolution of SARS‐CoV‐2, one might expect a higher prevalence of C > U mutations in the S protein. However, when we compared the frequency distribution of 12 substitution types within the S protein (Figure 2A) to that observed across the entire SARS‐COV‐2 genome, we found that while C > U mutations remained the most dominant substitution type, their frequency significantly decreased from 33.2% genome‐wide to 27.0% in the S protein (FE = 0.81, *p* = 2.8 × 10^−7^, Figure 2B). In fact, C > U, A > G, and G > A exhibited the lowest FEs among the 12 substitution types in the S proteins. In contrast, G > U mutations were enriched particularly in the S protein (15.8% vs. 14.8% genome‐wide, FE = 1.07, Figure 2B). The FE of C > U demonstrated an inverse pattern compared to the FE of G > U in many viral proteins, including N, ORF6, ORF8, ORF3a, and ORF1a/ORF1ab nsp3/nsp4/nsp5/nsp8 (Figure 2B).

We further examined the substitution frequencies in other functional regions and sites, such as IDRs and non‐synonymous mutation sites. Analysis of IDR sequences from the DistProt database revealed that C > U frequency in IDRs (26.7%, Figure 2C) was significantly lower than that across the genome (FE = 0.80, *p* = 2.1 × 10^−^^9^), along with other RNA editing‐related substitutions, such as A > G (11.6% vs. 13.7% genome‐wide, FE = 0.84, *p* = 0.007) and U > C (13.3% vs. 15.3% genome‐wide, FE = 0.87, *p* = 0.016) (Figure 2D). In contrast, G > U was significantly enriched in the IDRs with frequency of 17.8% (FE = 1.2, *p* = 4.0 × 10^−4^), in addition to C > G (FE = 2.4, *p* = 7.9 × 10^−4^), G > C (FE = 2.1, *p* = 2.6 × 10^−7^), and U > A (FE = 1.6, *p* = 5.4 × 10^−3^).

Focusing on non‐synonymous sites, we also observed lower frequencies of C > U (30.9%, FE = 0.93, *p* = 1.1 × 10^−7^) and U > C (6.3%, FE = 0.41, *p* = 3.5 × 10^−171^) mutations than the genome‐wide levels (Figure 2E,F). This effect was even more pronounced in non‐synonymous mutations of S protein (22.0% and 5.7% for C > U and U > C, respectively, with FE = 0.66 and 0.37, *p* = 2.3 × 10^−12^ and 1.9 × 10^−17^) and other proteins, including M, N, and ORF3a, for which G > U were more frequently observed (Figure 2E,F). These findings indicate that G > U mutations, rather than C > U, are more likely to occur in functional sites within the SARS‐CoV‐2 genome, suggesting a functional role in viral adaptation.

**Figure 3:**
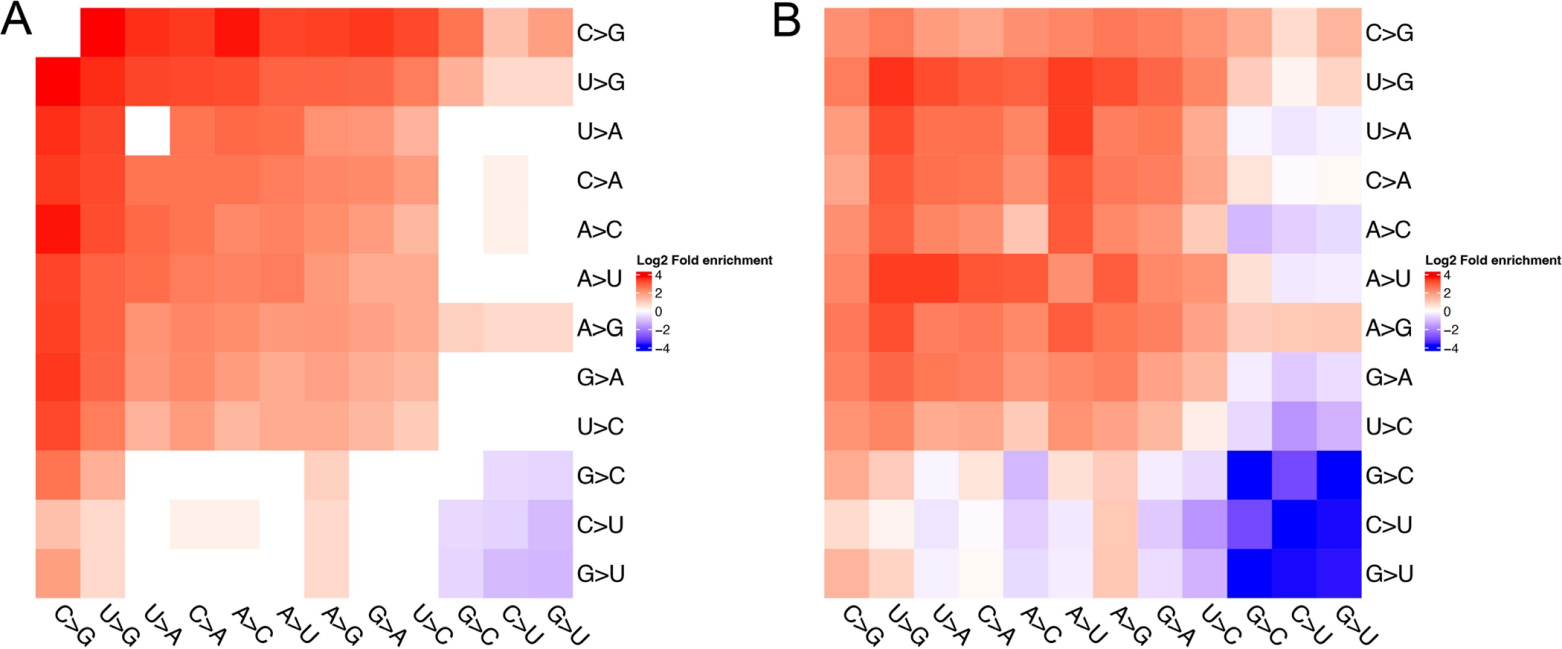
Concurrent mutations genome wide and within S protein only. (A) FE of concurrent pairs genome wide. (B) FE concurrent pairs in S protein only.

9963 SNVs formed 85 693 concurrent pairs with concurrent ratio above 0.9 (Equation 4). We conducted fold enrichment analysis (Equation 5) by comparing the observed concurrent pair frequency to the theoretical frequency (Equation 6). G‐loss mutations including G > U, G > A, and G > C tended not to pair with each other simultaneously (FE < 0.57, Figure 3A), instead, they preferentially co‐occurred with G‐gain mutations such as C > G and U > G (FE > 1.86, Figure 3A). Such trends were even more pronounced in the S protein (Figure 3B). For example, the FEs of G > U concurrent with G > U, G > A, and G > C decreased to 0.22, 0.04, 0.05 from 0.41, 0.56, and 0.51, respectively. In contrast, FEs of G‐loss mutations with A > G elevated to 2.18, 1.58, and 1.61, respectively. Concurrent pairs of C > U and themselves became depleted in the S protein (FE = 0.09, *p* = 4.6 × 10^−92^).

### 3.3 G > U Frequency Declined Sharply Since Omicron Emergence

The number of patient samples collected each month in the GISAID database fluctuated significantly during the COVID‐19 breakout through 2024, ranging from approximately 3000 to nearly 650 000. When comparing mutation frequencies across groups with uneven population size, for example, grouping samples by months, we found that groups with larger SNV pool sizes resulting from larger populations tended to exhibit lower C > U substitution frequencies. To prevent bias due to the sample size, we conducted random sampling to standardize SNV pool size in the way of randomly selecting a subset of samples from each population, ensuring that the mutation pool size was about 3550. This process was repeated 1000 times for each large size group through permutation.

**Figure 4:**
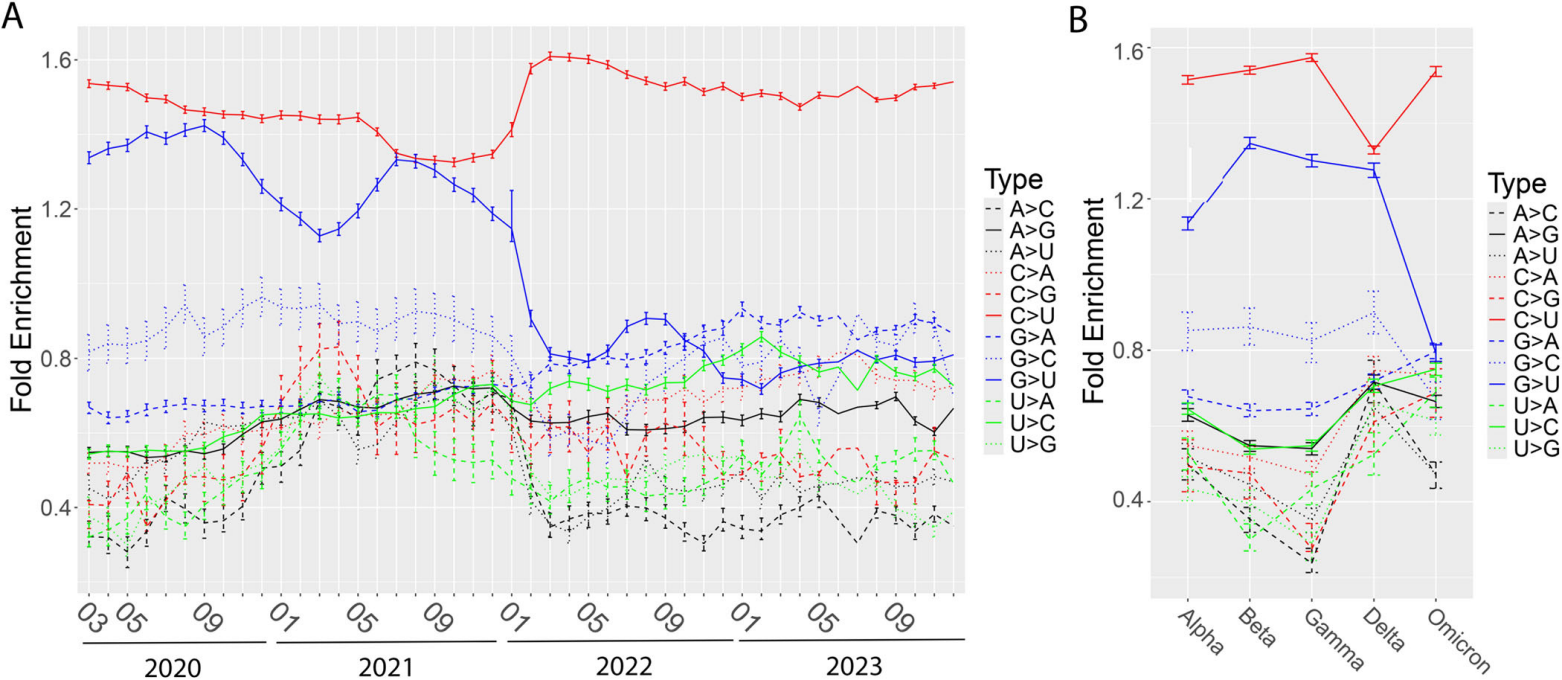
The FE of substitution types throughout the pandemic and across the prominent COVID‐19 variants of concern. (A) Twelve mutation FEs from March 2020 to January 2023. The FE was calculated using random sampling to control the SNV pool size (~3500). Random sampling was performed 1000 times for each scenario. The error bars at each point represent the 25th and 75th percentiles of FE values based on random sampling. (B) FEs of five major COVID‐19 variants using random sampling.

The median C > U frequency of 1000 simulations in each month remained above 36.7%, consistently higher than the general genome level (33.2%, Figure 4A). It started with the highest peak at 50.9% (FE = 1.5) at the beginning of the pandemic (2020–2023), then gradually dropped to 36.7% (FE = 1.1) by the end of 2021 (2021–2012). However, it surged again starting in January 2022 (Figure 4A). As a comparison, G > U increased slowly during the early stages of COVID‐19 pandemic, followed by decreases in the fall of 2020 till the spring of 2021. The FE of G > U matched the level of C > U during the summer of 2021 before declining again. Opposite to C > U substitution, G > U dropped sharply since 2022 with the emergence of Omicron (Figure 4A). We also assessed the patterns across five major COVID‐19 variants, including Alpha, Beta, Gamma, Delta, and Omicron (Figure 4B). The trends of the 12 substitution types were generally consistent with the time course patterns. Both C > U and G > U substitutions peaked in Beta with FE of 1.4 and 1.3, respectively. C > U exhibited its lowest median frequency (37.0%) and FE (1.1) with Delta but rebounded to 43.9% (FE = 1.3) in Omicron. In contrast, the FE of G > U continued to decline, dropping to 0.9 in Omicron, indicating a frequency lower than the genome level.

### 3.4 Substitutions of C > U and G > U in Different Immune Environments

Sequence data for 14 746 patients with known vaccine status were downloaded from the GISAID. To investigate whether the vaccination influences frequencies of different mutations, we selected 11 510 samples (2167 unvaccinated and 9343 vaccinated) that were collected within the same time frame (April 2021 to May 2022) with a roughly equal distribution across this period, to compare the vaccinated and unvaccinated groups. Additionally, we implemented a random sampling procedure 1000 times to downsize the vaccinated group to 2167 samples, matching the actual size of unvaccinated samples for every month.

**Figure 5:**
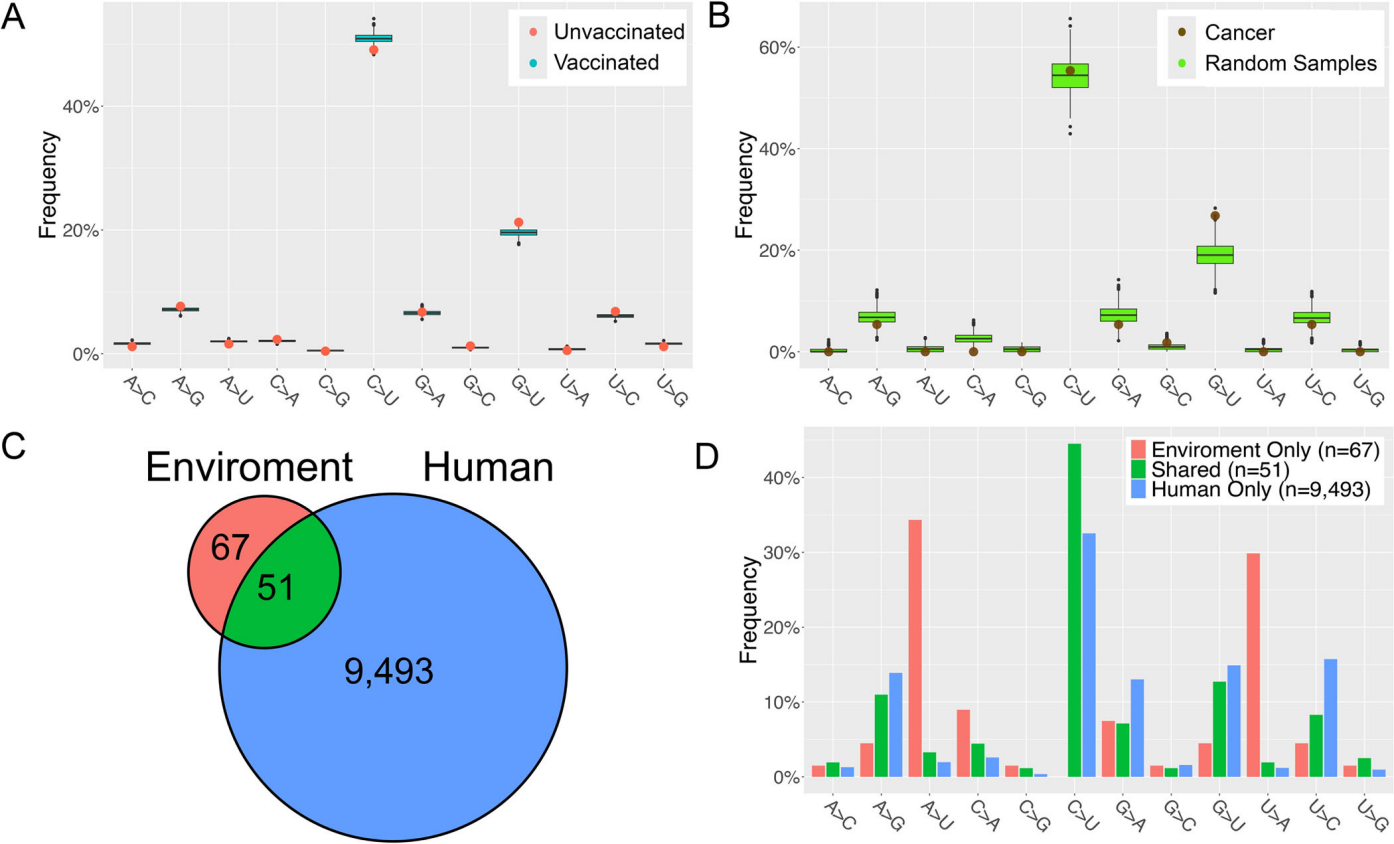
The distribution of SNVs of 12 substitution types in different immune environments. (A) Distribution of frequencies by random sampling in the vaccinated group to match the population size of unvaccinated group in each month. The red dot represents FEs of the unvaccinated group. (B) Comparison of transition frequencies between cancer patients and randomly sampled non‐cancer patients during the same time period. (C) Numbers of SNVs shared by human and environment hosts and exclusively identified in either human or environment. (D) The frequencies of 12 substitution types in SNVs categorized in (C).

As observed, the smaller sample sizes tend to result in higher frequencies of C > U and G > U. In the 2167 unvaccinated patients, C > U and G > U exhibited frequencies of 49.1% (FE = 1.48) and 21.2% (FE = 1.43), respectively (Figure 5A), compared to all samples (Figure 1A). But in randomly selected downsized vaccinated samples, C > U exhibited significantly higher frequency (50.9% ± 0.8% with FE = 1.54 ± 0.02 and *p* = 0.008) after 1000 times permutation. As contrast, G > U frequency in the vaccinated group decreased to 19.6% ± 0.6% (FE = 1.32 ± 0.04 and *p* = 0.002).

Intriguingly, among 48 COVID‐19 cancer patient samples collected between April and May 2020 [35], the frequency of C > U transition was 55.4%, while G > U mutations occurred at a rate of 26.8% (Figure 5B). To assess whether these mutation frequencies were significantly different from those in the general population, we conducted 1000 rounds of random sampling, ensuring the sample size of general patients collected during the same period matched that of the cancer group. The results (Figure 5B) revealed a nearly identical C > U mutations frequency of 54.4% (±3.5%). However, the frequency of G > U mutations in the general population was significantly lower, 19.1% ± 2.6%, with *p* = 0.001, indicating a notable enrichment of G > U transition in the cancer cohort.

We also compared 586 SNVs identified from 2724 environment samples with 10 012 SNVs in human samples. The majority (519) of environmental SNVs were also found in human samples as well, leaving 67 SNVs exclusive to the environment samples (Figure 5C). Interestingly, none of these 67 unique SNVs was C > U mutation. Instead, A > U and U > A mutations were more prevalent in SNVs unique to the environment (Figure 5D), while the frequencies of G > U, A > G, and U > C were lower.

## 4 Discussion

In this study, we utilized a large cohort of over 5 million complete SARS‐CoV‐2 genomes to examine the SNV patterns for 12 substitution types across various genomic regions such as individual genes, functional sites including IDRs and non‐synonymous mutations, time course, major COVID‐19 variants, and host settings. As anticipated, our findings confirmed previous reports, revealing that C > U mutations are the most prevalent, accounting for 33.2% of all SNVs, with a mutation ratio of 1.8 when normalized by the total numbers of reference nucleotides. This was followed by G > U and other RNA editing‐related substitution types including A > G, U > C, and G > A. These results underscore RNA editing as a major source of mutations in SARS‐CoV‐2, even though G > U, as the second most dominant mutation, is unusual for a transversion mutation and not typically associated with RNA editing.

However, C > U frequencies were lower in key functional regions, including the S protein, IDRs, and non‐synonymous sites, compared to the whole genome. In contrast, G > U showed the opposite pattern, being enriched in these functional regions with significantly higher frequencies than the genome‐wide level. Notably, among 1480 G > U mutations across the genome, 85.5% were nonsynonymous, and nonsynonymous G > U mutations in S protein (*n* = 184) outnumbered C > U mutations (*n* = 169), emphasizing its potential functional significance in SARS‐CoV‐2 evolution.

Before 2022, C > U and G > U frequency variations followed a similar trend, both gradually decreasing as SARS‐CoV‐2 evolved. However, after reaching its lowest point in the Delta variant, C > U frequency surged dramatically in early 2022, while G > U continued its decline throughout the pandemic. As the predominant substitution type, C > U is likely influenced by the host immune system. The vaccinated group exhibited a slightly but significantly higher C > U mutation frequency (*p* = 0.008) and a significantly lower G > U frequency (*p* = 0.002) compared to the unvaccinated group. These differences might be influenced by the variant compositions in each group, as the vaccinated group included a higher proportion of Omicron samples (9.2% vs. 4.9%) and lower rate of Delta samples (82.8% vs. 86.2%) than the unvaccinated group. Additionally, we observed a significantly higher frequency of G > U mutations in cancer patients compared to the general population during April to May 2020. Intriguingly, a comparison of human and environmental samples revealed that C > U mutations were absent in SNVs unique to environmental samples, along with other RNA‐editing‐related mutations such as A > G and U > C depleted as well. Instead, non‐RNA‐editing mutations like A > U and U > A were predominated, supporting the notion that C > U and other RNA editing mutations primarily originate in or are driven by the human host immune system.

G > U mutations are often linked to reactive oxygen species (ROS), as ROS activity on guanine causes its oxidation to 7,8‐dihydro‐8‐oxo‐2’‐deoxyguanine (oxoguanine), which mispairs with adenine (A) base, resulting in G > U substitutions [11, 36, 37]. Given that oxidative stress, responsible for excessive ROS production, plays an essential role in pathogenesis of COVID‐19 [38], the occurrence of G > U mutations, especially those on functional regions, may be associated with the severity of COVID‐19 symptoms. The decline in G > U mutations over the course of pandemic, particularly for Omicron, could be attributed to gradually developed population immunity and tolerance to SARS‐CoV‐2 infection, which mitigated symptom severity. Additionally, vaccination, which is known to reduce symptom severity [39, 40], may explain why the vaccinated group showed lower G > U mutation frequency than the unvaccinated group. When analyzing concurrent mutations, we found that G‐loss substitutions such as G > U, G > A, and G > C rarely appeared together. Instead, G‐gain mutations involving A, C, or U converting to G tended to more frequently co‐occur with others, especially in the S protein, suggesting a compensatory mechanism for the loss of G in G > U mutations.

Most COVID‐19 studies lacked comprehensive and well‐structured epidemiological designs, limiting robust conclusions. A key issue with currently available SARS‐CoV‐2 genome data is bias from inconsistent sample collections across different regions and time, leading to unbalanced sample sizes across different conditions, as outlined in our manuscript. This made it difficult to fairly assess the effects of confounding factors beyond the mutations themselves. Additionally, the evolving pandemic introduced complexity, as variants emerging at different times and in different populations, along with immune stress, may influence mutation patterns independently of direct biological effects, making comparisons, such as between vaccinated and unvaccinated individuals, more challenging. To better understand viral evolution, future research should use standardized data collection, detailed patient metadata, and improved statistical models to separate true mutation effects from confounding influences.

In summary, our study provides a comprehensive analysis of SARS‐CoV‐2 SNV patterns, emphasizing the predominant role of C > U mutations and the functional significance of G > U substitutions. The enrichment of G > U mutations in key functional regions, their steady decline over time, and their potential association with oxidative stress highlight their role in viral evolution and disease severity. The distinct substitution patterns between vaccinated and unvaccinated groups further underscore the influence of host immunity on SARS‐CoV‐2 mutational dynamics. The elevated frequency of G > U mutations in cancer patients suggests a possible connection to cancer‐associated factors, such as oxidative stress or modifications in the cellular microenvironments. These findings enhance our understanding of SARS‐CoV‐2 evolution, with implications for tracking viral adaptation, vaccine development, and therapeutic interventions. Future studies should investigate the mechanistic basis of G > U substitutions in oxidative stress response and their impact on viral fitness. G > U mutations in circulating viral strains may decrease the match between the vaccine‐encoded spike protein and circulating variants, resulting in reduced antibody neutralization and vaccine efficacy. Gaining deeper insights into these processes could help refine vaccine design strategies that specifically target G > U mutations, potentially mitigating disease severity even when the virus evades existing vaccines.

## Author Contributions

**Xierzhatijiang Sulaiman:** methodology, investigation, visualization, writing – original draft, writing – review and editing. **Yan Han:** methodology, investigation, visualization, supervision, writing – original draft, writing – review and editing. **Sheng Liu:** investigation, visualization, writing – review and editing. **Kailing Li:** investigation, visualization, writing – review and editing. **Marissa Shang:** methodology, investigation, visualization, writing – original draft, writing – review and editing. **Lei Yang:** investigation, supervision, funding acquisition, writing – review and editing. **Kenneth White:** investigation, project administration, funding acquisition, writing – review and editing. **Yong Zang:** conceptualization, methodology, investigation, supervision, funding acquisition, writing – review and editing. **Jikui Shen:** conceptualization, methodology, investigation, visualization, project administration, supervision, writing – original draft, writing – review and editing. **Jun Wan:** conceptualization, methodology, investigation, visualization, project administration, supervision, funding acquisition, writing – original draft, writing – review and editing. All authors read and approved the final manuscript.

## Conflicts of Interest

The authors declare no conflicts of interest.

## Data Availability

The data that support the findings of this study are available from GISAID. Restrictions apply to the availability of these data, which were used under license for this study. The analyzed SNV data will be provided upon request and in accordance with the agreement with GISAID.
